# Theta Transcranial Alternating Current Stimulation Over the Dorsomedial Prefrontal Cortex Does Not Enhance Long‐Term Memory

**DOI:** 10.1111/ejn.70431

**Published:** 2026-02-10

**Authors:** Dima Chitic, Krasimir S. Zdravkov, Vasiliki Dounavi, Mark R. Nieuwenstein, Miles Wischnewski

**Affiliations:** ^1^ Department of Psychology University of Groningen Groningen the Netherlands; ^2^ Research School for Behavioural and Cognitive Neurosciences University of Groningen Groningen the Netherlands

**Keywords:** dorsomedial prefrontal cortex, long‐term memory, theta oscillations, transcranial alternating current stimulation

## Abstract

Long‐term memory (LTM) has been associated with neural oscillation in the theta (3–8 Hz) range. Although previous studies have suggested that the dorsomedial prefrontal cortex (dmPFC) is a core region for LTM retrieval, causal evidence is sparse and mixed. Furthermore, the moderating effects of stimulus memorability have not yet been explored. In the present study, we used transcranial alternating current stimulation (tACS) to modulate theta oscillation in the dmPFC during the retrieval of visual images with varying levels of memorability. Specifically, we included *n* = 33 healthy volunteers who were exposed to 300 images of faces, scenes and items, which they had to memorize. Recognition accuracy was assessed 1 h later. During the retrieval phase, participants received either sham or verum (4 Hz, 2.5 mA) tACS and were asked whether they had seen the pictures before (150 new and 150 old). Contrary to our preregistered hypotheses, we found no significant effect of 4‐Hz tACS applied during retrieval on LTM recognition. Furthermore, although the memorability effect was observed, it did not interact with tACS, indicating that stimulation neither improved nor worsened performance on low‐ and high‐memorable images. Altogether, the present study does not support an active role of 4‐Hz oscillations in the dmPFC for the recognition of images with varying levels of memorability, under the specific task and stimulation parameters used here. However, this null effect may be specific to the task and particular parameters used in this study.

## Graphical abstract

We tested whether 4‐Hz transcranial alternating current stimulation over the dorsomedial prefrontal cortex enhances long‐term memory retrieval. Thirty‐three participants encoded images of faces, objects and scenes varying in memorability and completed a recognition test during stimulation. Theta tACS did not improve memory performance for any stimulus type or memorability level.

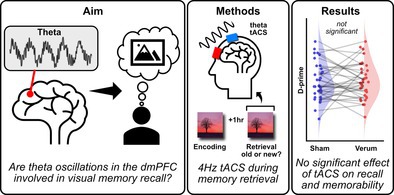

**Abbreviations**
BF: Bayes factor
CI: confidence interval
D′: D prime
dmPFC: dorsomedial prefrontal cortex
EEG: electroencephalography
Hz: Hertz
LTM: long‐term memory
M: mean
SE: standard error of mean
tACS: transcranial alternating current stimulation

## 1 Introduction

Long‐term memory (LTM) in humans refers to the storage of information acquired through experience, with new information being added through the sequential processes of encoding and consolidation and being subsequently accessed through retrieval (Squire et al. 1993; Squire and Wixted 2011; Guskjolen and Cembrowski 2023). Memory can be declarative or nondeclarative, depending on whether it is retrieved explicitly, like in verbal recall of technical knowledge, or implicitly, like in motor skills. A subset of declarative memory is episodic memory, which comprises encoded events with temporal and spatial associations (Squire and Wixted 2011). Essentially, episodic memory is the storage of one's life events.

However, we are not equally likely to remember all the events or stimuli that we experience, as some inputs are more memorable than others (Bainbridge et al. 2013, 2017; Isola et al. 2014). Memorability refers to an intrinsic and stable quality of images, such that, on average, one tends to recognize some pictures better than others (Isola et al. 2014). This memorability effect seems to be preserved across individuals, as evidenced by significant correlations among participants in the memorability of images (Isola et al. 2014; Bainbridge et al. 2017). Although memorability is often studied on relatively short timescales (within one continuous session), there are also studies demonstrating that it similarly affects picture recognition on a longer timescale of days in a classic LTM paradigm (Goetschalckx et al. 2018). Therefore, it is an essential factor to control for and assess in studies of LTM.

The neural mechanisms of both LTM and memorability have been studied over the past decades, highlighting an important role of neural oscillations. In particular, oscillations in the theta frequency (3–8 Hz) have been associated with various memory aspects, including encoding, active maintenance and retrieval (Herweg et al. 2020). Due to its slow waveform, theta frequency is commonly hypothesized to play a part in long‐range connectivity among populations of neurons (Singer 1993; Colgin 2013), resulting in various cortical and subcortical regions displaying memory‐related theta. Based on both animal and human studies, these regions include the hippocampus, medial temporal cortex, lateral and medial prefrontal cortex, and parietal cortex (Buzsáki 2002; Lega et al. 2012). This interregional coordination at the theta frequency has been particularly shown to underlie the communication between the hippocampus and the neocortex in learning and memory processes (Kim et al. 2011; Gattas et al. 2023).

Nyhus and Curran (2010) proposed that cortical theta is responsible for temporally ordering episodic events. Theta oscillations related to LTM are found in various cortical regions, including prefrontal areas (dorsolateral, ventrolateral and ventromedial prefrontal cortices), temporal lobes, posterior parietal regions and even occipital areas (Guderian et al. 2009; Klink et al. 2020; Grover et al. 2022; Luckey et al. 2022; Rolls 2022; Manippa et al. 2024; Sun et al. 2025). Another region of interest is the frontal midline, encompassing the dorsomedial prefrontal cortex (dmPFC) and the dorsal anterior cingulate cortex (dACC). These regions are likely the origin of frontal midline theta oscillations (Tsujimoto et al. 2006; Cavanagh et al. 2012; Cavanagh and Frank 2014) and have been associated with cognitive control and, hence, a variety of processes, including reward learning, performance monitoring, working memory and attentional control (Cohen 2011; Hsieh et al. 2011; Enriquez‐Geppert et al. 2014; Hou et al. 2025; Surrey et al. 2025). Crucially, midfrontal theta power has been observed to be modulated during successful memory encoding and retrieval (Hanslmayr et al. 2010; Staudigl et al. 2010; Hsieh and Ranganath 2014; Pastötter and Bäuml 2014; Backus et al. 2016; Gattas et al. 2023). Rodent studies have shown that the medial prefrontal cortex is implicated in the recall of episodic memories by directing the hippocampus to retrieve context‐appropriate memories (Miller and Cohen 2001). Furthermore, in a human electroencephalography (EEG) study, Pastötter and Bäuml (2014) showed that episodic retrieval is positively correlated with slow midfrontal theta power (3–5 Hz), which predicted successful remembering. In contrast, fast (6–8 Hz) midfrontal theta power was associated with decreased memory performance.

Besides observational methods like EEG, others have used neuromodulation methods to alter oscillatory rhythms and investigate the link to LTM. Transcranial alternating current stimulation (tACS) is of particular interest as it can entrain neural activity (Krause et al. 2019; Wischnewski, Tran, et al. 2024) and thereby modulate oscillatory power (Helfrich et al. 2014; Kasten et al. 2016; Wischnewski et al. 2019). Specifically, tACS applies a low‐intensity alternating current to the head, part of which reaches the cortex and modulates neural activity at the stimulation frequency (Elyamany et al. 2021; Wischnewski et al. 2023; Agboada et al. 2025). Furthermore, tACS at 3 Hz over midfrontal regions has been shown to improve both free recall and multiple‐choice test performance (Shtoots et al. 2024, 2025). Moreover, theta tACS targeting medial frontal regions has been shown to improve memory in elderly volunteers with subjective memory impairments (Varastegan et al. 2023). However, Ergo et al. (2020) found no improved performance on declarative memory after dmPFC tACS at 6 Hz. Furthermore, it has been suggested that tACS is more effective if baseline scores are low (i.e., when there is more room for improvement). For instance, Grover et al. (2022) showed stronger tACS‐related effects in participants with lower baseline scores.

Altogether, there is mixed evidence that midfrontal theta tACS may improve LTM. However, various experimental designs, tasks and stimulation protocols have been used across various studies. Furthermore, the effect of memorability in tACS modulation of LTM is currently unknown. Therefore, the present preregistered study (please see https://aspredicted.org/nrjz‐qqx8.pdf) investigated the effects of theta tACS over the dmPFC on LTM for face, object and scene images, while also examining potential interactions with picture memorability. Our main hypothesis was that 4‐Hz tACS over dmPFC improves retrieval accuracy compared with a sham stimulation control condition. Second, we hypothesized that the effect of tACS is moderated by memorability, such that low‐memorable items would show a larger improvement with tACS than high‐memorable items. Although, to our knowledge, there is no prior work directly examining tACS effects as a function of stimulus memorability, this hypothesis follows from the idea that memorability reflects baseline memory strength. Low‐memorability items are more weakly represented and thus offer greater potential for neuromodulatory enhancement, analogous to baseline‐dependent effects observed in prior tACS studies (Grover et al. 2022). Finally, we postulated an exploratory hypothesis that tACS affects the three different picture categories differently. To test our hypotheses, we assessed the 1‐h retrieval performance of visual images from three verified databases (Bainbridge et al. 2013; Lu et al. 2020; Kramer et al. 2023). TACS location was determined based on meta‐analytic evidence (Chitic and Wischnewski 2025). Altogether, the goal of the present study was to elucidate the role of theta oscillations from the dmPFC in memorability and LTM.

## 2 Methods

### 2.1 Participants

Forty‐three volunteers were recruited. After excluding those who did not attend the second session (*n* = 8) or whose performance deviated by more than 2.5 absolute deviations from the median (*n* = 2), 33 participants remained (19 female and 14 male, *M*_*age*_ = 21.24, SD = 3.06). To a large extent, participants were recruited through the online participant pool used by the University of Groningen, with psychology students comprising the majority of participants. Participants could only take part in the study if they satisfied all of the inclusion criteria: absence of neurological, psychiatric, memory‐related or skin diseases; no history of epileptic episodes; no tattoos or piercings on the scalp; and no metallic plate in the head or any kind of stimulator in the whole body. As stated in the preregistration (please see https://aspredicted.org/nrjz‐qqx8.pdf), a power analysis suggested 32 participants to achieve a power of 0.80 at a significance level of 0.05 and a presumed effect size of 0.52, with the latter derived in GPower from a medium effect size of 0.4 and a group correlation of 0.7 (Faul et al. 2009; Brysbaert 2019). The study was approved by the local ethics board of the Faculty of Behavioural and Social Sciences of the University of Groningen. All participants provided written informed consent prior to participation.

### 2.2 Long‐Term Memory Task

**FIGURE 1:**
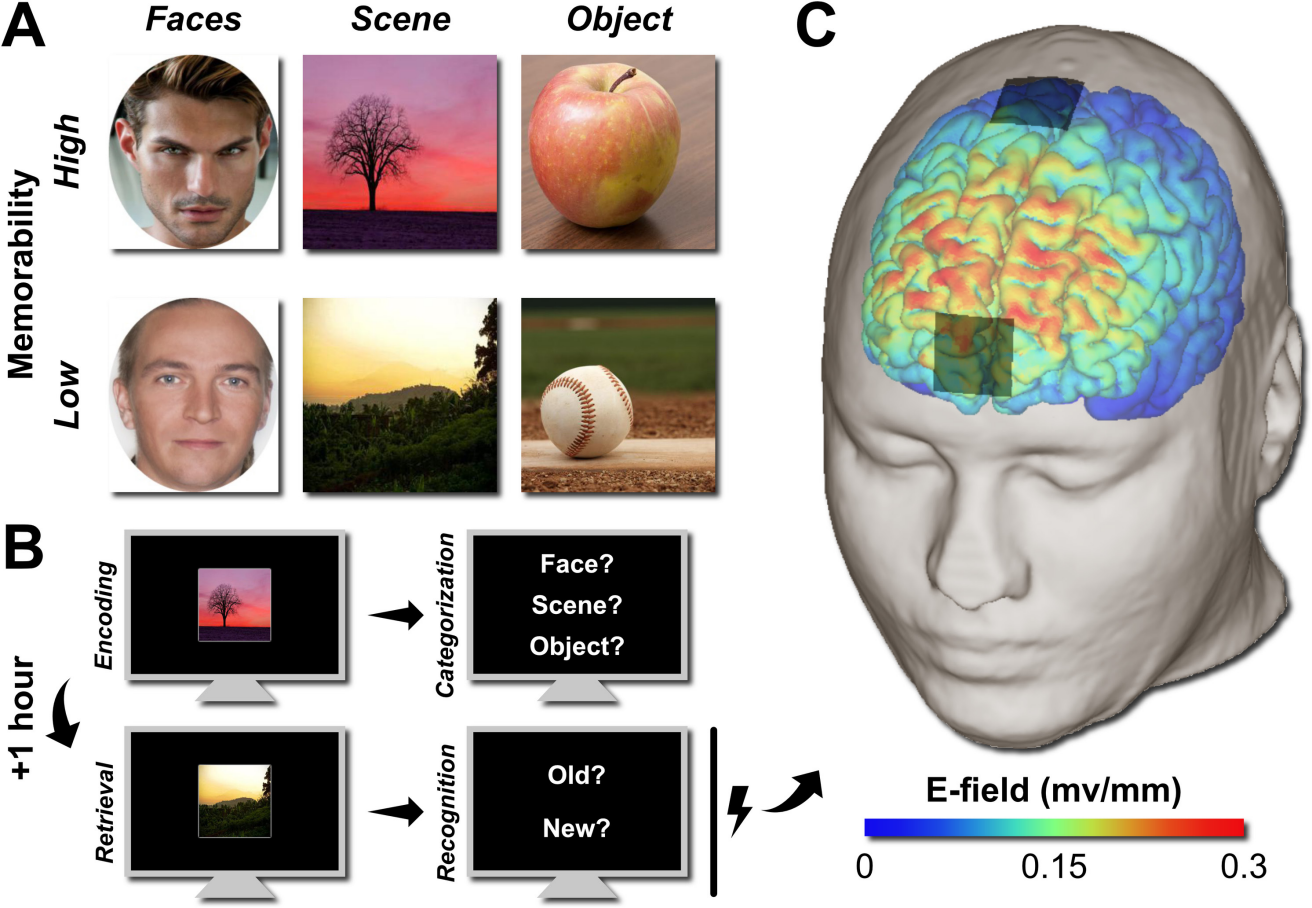
Stimuli, experiment and stimulation protocol. (A) Examples of stimuli used in the experiment. (B) During the encoding phase, participants were asked to memorize 300 images (100 per category and 150 per memorability level). To ensure participants remained attentive, they were asked to indicate the image's category. During retrieval, participants saw 150 images from the encoding phase and 150 new images. Participants were asked to indicate whether the presented image was old or new. (C) During retrieval, 4‐Hz tACS was applied at an intensity of 2.5‐mA peak to peak, targeting the dorsomedial prefrontal cortex. Electric field distributions are shown, and the electric field strength is expressed in mV/mm.

The stimuli for the task were taken from three different memorability databases, of which one contained pictures of natural scenes, another one of faces and the third one of objects (Bainbridge et al. 2013; Lu et al. 2020; Kramer et al. 2023). From each database and its corresponding category, we selected 160 pictures with the highest memorability and 160 with the lowest, for a total of 960 pictures. The object pictures were filtered by filename so that no two pictures of the same object were included, but similar objects were included (e.g., two pigs were not included, but a pig and a piglet were). The pictures in the object database were also screened to exclude fully visible faces or inappropriate content (e.g., a close‐up of a loincloth). Finally, all pictures were resized to 384 × 384 pixels, except faces, which were 384 pixels high and retained their width‐height ratio. Overall, the resulting stimuli could be divided into six groups based on two factors: category (face, scene and object) and memorability (low and high). Examples are shown in Figure 1A.

During encoding, participants were shown 300 pictures (50 per group), each presented for 1.5 s, with a jittered interstimulus interval of 1.5 ± 0.15 s, whereas a fixation dot was displayed. We explicitly instructed participants to memorize the pictures. To ensure that participants paid attention to the stimuli during encoding, they had to categorize them into scenes, faces or objects by pressing one of three keyboard buttons (A, W or D, respectively), as shown in Figure 1B. The average accuracy of categorization was (mean ± SE) 91.4% ± 1.4%. Before starting the task, they had 12 practice trials. The retrieval task was performed 1 h after the encoding task. During the retrieval task, 300 pictures were presented at the same image and interstimulus durations, with half drawn from the encoding phase and the other half new (not previously seen). Participants pressed one of two buttons to indicate whether the picture was old or new (A or D, respectively), as shown in Figure 1B. Both tasks contained two 20‐s breaks after 100 and 200 pictures were shown.

### 2.3 tACS

A NeuroConn DC Stimulator Plus was used to apply tACS. Neuromodulation was administered during the retrieval phase, with the two conditions (sham and verum) counterbalanced across the two sessions. We targeted the dmPFC using a meta‐modelling method that predicts the optimal stimulation location by combining evidence from electric field models and behavioural effect sizes (Wischnewski et al. 2021; Wischnewski, Berger, et al. 2024). Specifically, data from *N* = 17 previous theta tACS studies were used to identify the optimal target location (Figure S1). Note that at the time of starting the current study, the dmPFC target was based on preliminary data and that the completed meta‐modelling study is published elsewhere (Chitic and Wischnewski 2025). To target the dmPFC, two conductive rubber electrodes covered with Ten20 adhesive paste were positioned halfway between AFz and Fpz and halfway between Cz and FCz, respectively. The electrodes were 3 × 3 cm each, and the impedances were kept below 10 kΩ. A sinusoidal current at a frequency of 4 Hz (theta) and a current strength of 2.5‐mA peak to peak were applied. The lower end of the theta range was chosen, as it has previously been suggested to relate to improved memory retrieval (Pastötter and Bäuml 2014). The corresponding electric field distribution was modelled in SimNIBS on a standard head model (Figure 1C). The participants were briefly habituated to tACS before the retrieval task, using current strengths of 0.75, 1.5, 2 and 2.5 mA in both sessions, each applied for a few seconds after a 30‐s ramp‐up. The verum stimulation also consisted of 30 s of ramp‐up, 30 s of ramp‐down and 20 min of 2.5 mA stimulation in between, for a total of 21 min. The sham stimulation lasted 30 s, with ramp‐up and ramp‐down of 30 s each. The participants, but not the researchers, were blind to the stimulation condition. To verify blinding effectiveness, participants completed poststimulation questionnaires.

### 2.4 Procedure

The experiment consisted of two sessions, both held at the same time of day and scheduled 7–14 days apart, except for one participant, who attended the second session 1 month after the first. The two sessions followed the same procedure, except for the stimulation condition and the picture sets used. At the first session, participants were informed in writing and verbally about the experiment's purpose, outline and exclusion criteria, after which they signed informed consent and provided demographic data. After encoding, participants had a 40‐min break, during which they had to remain within the university building, keep their hair dry and refrain from consuming any psychoactive substances (e.g., coffee and cigarettes). After the break, tACS electrodes were placed on the participant's head, and brief habituation stimulations were applied. Before the start of the retrieval task, tACS (in the verum condition) was applied for 3.5 min and lasted until the end of the task. Finally, they completed the poststimulation questionnaire regarding the side effects they experienced (Antal et al. 2025). After the second session, the questionnaire also asked them to guess which session was a placebo. One session lasted approximately 2 h, including breaks.

### 2.5 Data Analysis

Memory performance was quantified with D‐prime (D′) and average reaction time. D′ is a metric derived from the signal detection theory, calculated as the difference between the standardized hit rate and the false alarm rate: *z* (hit rate) − *z* (false alarm rate) (Snodgrass and Corwin 1988). A repeated‐measures ANOVA (RM‐ANOVA) was used to investigate all three hypotheses, with D′ as the dependent variable, and tACS, category and memorability as within factors. These were accompanied by Bonferroni‐corrected post hoc multiple comparisons for groups of interest. Additionally, an exploratory Bayesian model was used to better interpret the main tACS effects, as well as the interactions between tACS and memorability and picture category, respectively. Bayes factors (BF_10_) were computed using the default prior implemented in JASP. Following conventional guidelines, BF_10_ values larger than 3 were interpreted as evidence for the alternative hypothesis, values smaller than 1/3 as evidence for the null hypothesis and values between 1/3 and 3 as inconclusive (Kass and Raftery 1995; Wetzels et al. 2011). The analyses were performed in R Studio and JASP.

## 3 Results

**FIGURE 2:**
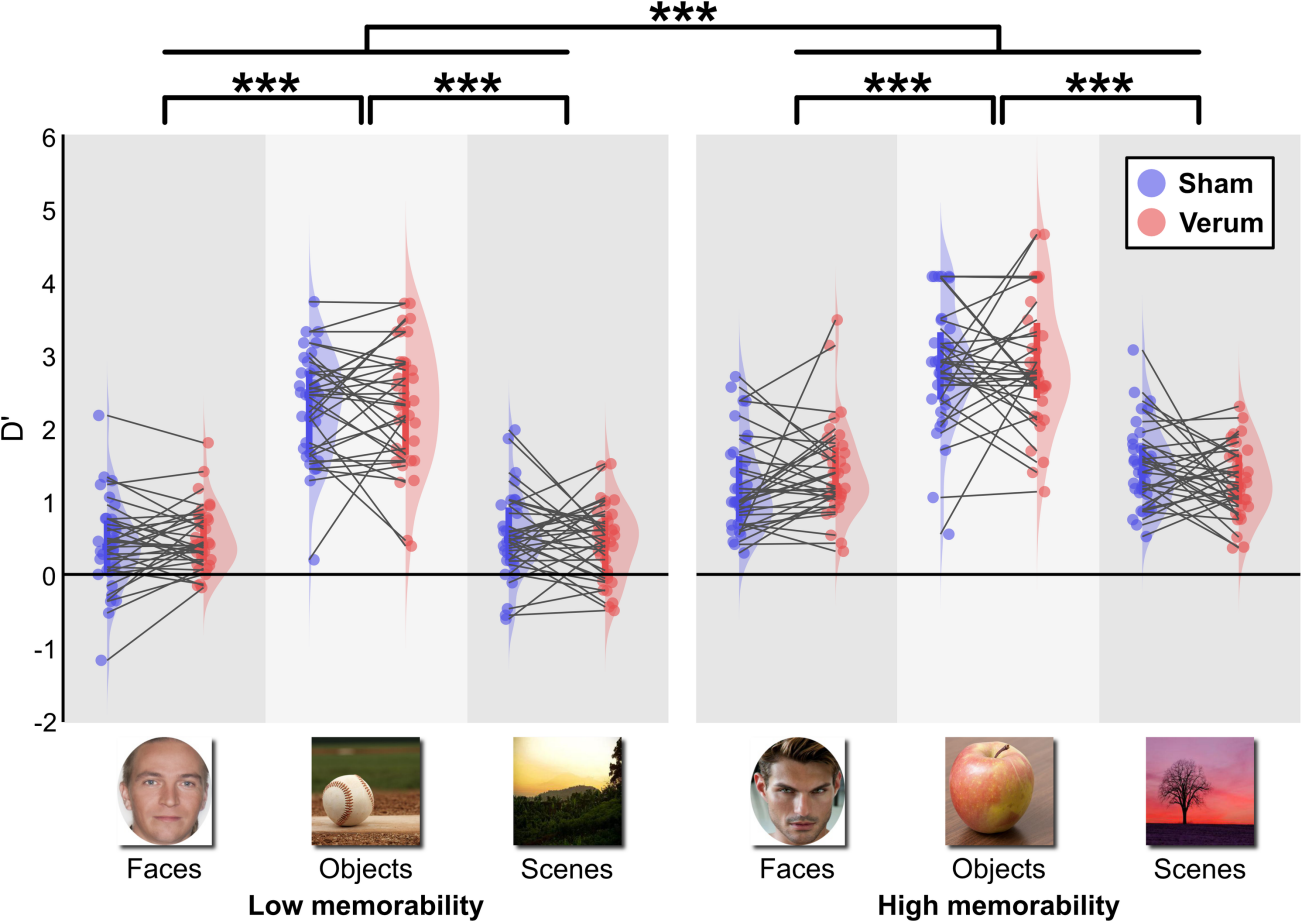
Recognition performance expressed in D prime (D′), separated for high and low memorability and stimulus categories, compared between sham and verum tACS.

As shown in Figure 2, real and sham stimulation did not differ significantly in memory performance (*F*[1, 32] = 0.44, *p* = 0.512, *M* = 0.05, SE = 0.08, 95% CI [−0.10, 0.20]). Memorability showed a robust effect (*F*[1, 32] = 358.86, *p* < 0.001), with more memorable pictures leading to better recognition (*M* = 0.79, SE = 0.04, 95% CI [0.70, 0.87]). Picture category also showed a significant main effect (*F*[2, 64] = 211.33, *p* < 0.001). Post hoc analysis showed that there was no difference in remembering scenes and faces (*M* = 0.10, SE = 0.10, 95% CI [−0.14, 0.33], *p* = 0.961), but objects were better remembered than both scenes (*M* = 1.65, SE = 0.10, 95% CI [1.42, 1.89], *p* < 0.001) and faces (*M* = 1.75, SE = 0.10, 95% CI [1.51, 1.98], *p* < 0.001). The interaction between tACS and picture category was not significant (*F*[2, 64] = 3.117, *p* = 0.051) nor was the interaction between tACS and memorability (*F*[1, 32] < 0.01, *p* = 0.987). However, there was a significant interaction of memorability and picture category in terms of memory performance (*F*[2, 64] = 6.58, *p* = 0.003). Specifically, there was a larger effect of memorability on faces (*M* = 0.88, SE = 0.08, 95% CI [0.64, 1.12], *p* < 0.001) and scenes (*M* = 0.94, SE = 0.08, 95% CI [0.70, 1.18], *p* < 0.001), whereas there was a smaller effect on objects (*M* = 0.54, SE = 0.08, 95% CI [0.30, 0.78], *p* < 0.001). When separately examining the hit rate and false alarm rate, we observed similar effects and no tACS effects (Figures S2 and S3).

An exploratory Bayesian analysis was performed to verify whether we could argue for a null effect of tACS on memory performance. The analysis yielded BF_10_ = 0.124, indicating evidence for the null hypothesis (no difference between sham and verum tACS). Similarly, for the interaction between tACS and memorability, evidence towards the null hypothesis was found (BF_10_ = 0.186). For the interaction between tACS and picture category, no conclusive evidence towards either the null or the alternative hypothesis was found (BF_10_ = 1.118).

To confirm that the behavioural null effect was not driven by differences in reaction speed, we also investigated reaction times during retrieval. Reaction times were similar between verum and sham tACS (*M* = 1.85, SE = 9.12, 95% CI [−16.29, 20.00], *p* = 0.840). This was also confirmed by an exploratory Bayesian analysis yielding BF_10_ = 0.190.

Lastly, the assessment of side effects indicates that phosphenes were experienced only in the verum condition (9%), but some degree of itching or hotness was reported in both verum (79%) and sham (30%) conditions. A Wilcoxon signed‐rank test of side‐effect severity indicated significantly more side effects and greater severity in the verum condition (*p* < 0.001). The proportion of participants who correctly guessed which condition was placebo (70%) was significantly different from chance (50%) according to a binomial test (*n* = 23, *N* = 33, *p* = 0.04).

## 4 Discussion

In the present study, we investigated the role of frontal midline theta stimulation during retrieval in LTM image recognition. Contrary to our preregistered hypotheses (please see https://aspredicted.org/nrjz‐qqx8.pdf), we found no effect of 4‐Hz tACS over the dmPFC on memory performance, with a nonsignificant result in a frequentist RM‐ANOVA and evidence in favour of the null hypothesis in a Bayesian model. Similarly, no interaction effects were observed, indicating that the stimulation effect did not differ across memorability levels or image categories.

The present results indicate that tACS, as applied here, is not effective in modulating LTM within the set of experimental design choices made. Indeed, research on LTM modulation takes various forms, and there is considerable variability in methodological approaches. Among others, important factors to consider are (I) stimulation frequency (e.g., low theta or high theta), (II) stimulation location, (III) timing of stimulation (during encoding, during retrieval, during both or in between), (IV) the LTM outcome measure (e.g., recognition, recall or semantic memory) and (V) modality of LTM stimuli (e.g., pictures, words or sounds). Within this multidimensional design space, our null results suggest that 4 Hz (I) tACS over dmPFC (II), during retrieval (III), is not effective in modulating recognition (IV) of visual images (V).

Taking into account, the multidimensionality of methodological choices may explain the contrast to previous findings. For example, Shtoots et al. (2024, 2025) reported increased LTM performance after midfrontal theta tACS. Although the stimulation location was similar between our study and theirs, several other factors differed. Shtoots et al. used a different task, different items and various memory assessments. They used either 30 object pictures (Shtoots et al. 2024) or immunology knowledge given over the span of a 15‐min lecture (Shtoots et al. 2025), meaning that both could be verbally rehearsed, unlike most of our 300 pictures. Indeed, early consolidation relies on the repeated activation of the memory trace soon after encoding (Guskjolen and Cembrowski 2023), which was less likely in our study. Furthermore, Shtoots et al. used free recall or multiple‐choice test performance as outcome measures, whereas the present study focused on recognition memory. Free recall requires uncued and self‐initiated retrieval and contextual reinstatement processes, whereas recognition tasks test (passive) familiarity. It is therefore likely that recall relies more on (medial) prefrontal control regions than recognition (Wheeler et al. 1997; Ranganath and Ritchey 2012), providing one potential reason for the discrepancy in results. The multiple‐choice testing can rely mostly on recognition or mostly on retrieval, depending on how plausible the alternative choices are (Bjork et al. 2015). Nonetheless, it is likely that they would lead to more effortful conscious retrieval than the paradigm used here, similarly showing that task variety could be a crucial difference between the studies.

The timing of the applied stimulation is also crucial in determining which memory process is targeted. In our study, the retrieval phase was targeted. In contrast, Shtoots et al. (2024, 2025) stimulated the dmPFC during the resting period between encoding and retrieval to enhance consolidation. Another study applying theta (6‐Hz) tACS over the dmPFC instead stimulated during the encoding of a word‐association task and found no beneficial effects compared to sham (Ergo et al. 2020). Therefore, if the timing of theta tACS over dmPFC is taken in isolation from the other variability factors, it suggests that an offline stimulation following encoding, in the period of consolidation, is most promising. The absence of effects in the study by Ergo et al. and in our study may relate to the isolation of encoding and retrieval stimulation. One account of memory retrieval proposes that neural activity patterns engaged during encoding are (partially) reinstated during successful retrieval (Johnson et al. 2009; Wimber et al. 2012). In line with this view, Javadi et al. (2017) applied tACS during both encoding and retrieval, either at matched or mismatched frequencies. They observed improved long‐term memory performance only when stimulation frequencies at encoding and retrieval matched, whereas no effect was observed for mismatched frequencies. Notably, this study applied stimulation at the gamma frequency over the left dorsolateral prefrontal cortex. If such context matching paradigms are equally effective for theta stimulation or for dmPFC remains to be determined by future research.

The rationale for targeting the dmPFC in this study was based on preliminary meta‐analytic results of *N* = 17 studies that applied theta tACS to modulate LTM (Figure S1). By combining observed behavioural changes with electric‐field modelling, we generated a map of where theta tACS correlated with improved memory performance (Wischnewski et al. 2021; Wischnewski, Berger, et al. 2024). Two important caveats should be noted about this approach. First, the included studies used a variety of experimental designs and stimulation protocols. For instance, this meta‐analysis included stimulation during encoding, retrieval and both. Furthermore, a variety of different tasks were employed. Second, the analysis was based on preliminary data that were available before the start of the present experiment. The full dataset, comprising *N* = 20 studies, shows a similar pattern, with a positive association between dmPFC theta tACS and LTM performance (Chitic and Wischnewski 2025). However, this association, although numerically the strongest, was statistically nonsignificant. Nevertheless, targeting the dmPFC in this study was informed by the available evidence at study onset. However, such aggregate findings cannot determine whether any specific task, timing or stimulation configuration will engage that region's contribution to memory retrieval.

As anticipated, we confirmed that more memorable pictures were remembered better than less memorable pictures (Bainbridge et al. 2013; Kramer et al. 2023). However, in our study, tACS did not differently affect items with differing memorability levels. This aligns with previous observations that memorability is a feature that is mostly processed in higher visual areas and the medial temporal lobe (Bainbridge et al. 2017; Bainbridge and Rissman 2018; Wang et al. 2025). Indeed, in an LTM experiment with high and low memorable items, Bainbridge et al. (2017) found activity in both the medial temporal and prefrontal cortex. However, whereas medial temporal lobe activity was modulated by memorability, prefrontal activity reflected individual differences in memory performance. As such, the absence of changes in memorability after theta tACS over dmPFC is not surprising.

In addition to memorability, a difference in LTM performance was observed across picture categories, with better performance for objects than for faces and scenes. This observation is likely a consequence of using different databases rather than a genuine preference for objects. Indeed, previously, it has been shown that recognition memory for faces is higher than for scenes at both short and long retention intervals (Keightley et al. 2011; Sato and Yoshikawa 2013). Crucially, no interaction between picture category and tACS was observed, consistent with the idea that the dmPFC is associated with domain‐general cognitive control processes (Cavanagh and Frank 2014). However, given the near‐threshold *p* value (*p* = 0.051), we cannot conclusively rule out a subtle category effect, which should be addressed in future work.

Several limitations of this study should be addressed. First, although targeting the dmPFC was motivated by preliminary meta‐analytic evidence (Figure S1), the stimulation focality of the present montage was limited. Besides the dmPFC, parts of the dorsolateral prefrontal cortex were stimulated. Based on our updated meta‐analysis (Chitic and Wischnewski 2025), stimulation of the lateral portions of the prefrontal cortex may worsen LTM performance. As such, a more focal multielectrode montage may have had different effects. Note that this negative association was not observed in the preliminary meta‐analysis that was used to determine stimulation montage for the present study (Figure S1). Second, our study contained no electrophysiological measures to quantify tACS modulation of theta oscillations. Given that the induced electrical current strength varies between individuals, the neurophysiological effects would likely have differed between individuals. As such, our study was not sensitive to any nonlinear relationships between induced electric fields and behavioural changes. Third, the same stimulation frequency of 4 Hz was used for all participants, not considering individual differences in peak theta frequency. Personalized stimulation parameters have been shown to enhance the effects of tACS (Hoornweder et al. 2025). However, given the moderate evidence in favour of the null hypothesis, we believe it is unlikely that individualized theta stimulation would have drastically changed the conclusions of our study. Fourth, the sample size for this study was adequate for detecting moderate, but not small, effects. The main hypothesis (comparing verum and sham tACS) would likely not have changed with a higher sample size, as indicated by the observed Bayes factor of 0.124. However, more marginal effects, such as the interaction between tACS and picture category (with a *p* value of 0.051 and a Bayes factor of 1.118), may have benefited from a larger sample size. Finally, we found that participants could distinguish verum from sham stimulation. Given the tACS null result, it is unlikely that this observation affects the interpretation of our results. However, it emphasizes that ramp‐up/down protocols, which are often used in tACS research, are not always sufficient for successful blinding (Turi et al. 2019; Neri et al. 2020; Sheffield et al. 2022).

Overall, we may conclude that theta tACS over the dmPFC, applied during the retrieval phase, is ineffective in modulating visual recognition of pictures of objects, scenes and faces. This does not imply that the dmPFC is entirely unrelated to LTM, but it may be more relevant to other aspects, such as memory consolidation or integration. However, given the specific experimental procedures used here, tACS appears ineffective at modulating memory performance or sensitivity to memorability.

## Author Contributions

**Dima Chitic:** formal analysis, investigation, methodology, writing – original draft. **Krasimir S. Zdravkov:** investigation, writing – review and editing. **Vasiliki Dounavi:** investigation, writing – review and editing. **Mark R. Nieuwenstein:** supervision, writing – review and editing. **Miles Wischnewski:** conceptualization, formal analysis, funding acquisition, methodology, supervision, writing – review and editing.

## Funding

This work was supported by the Nederlandse Organisatie voor Wetenschappelijk Onderzoek (406.XS.25.01.035).

## Conflicts of Interest

The authors declare no conflicts of interest.

## Supporting information


**Figure S1:** Meta‐analytic evidence (*N* = 17) on optimal stimulation location for theta tACS related to long‐term memory (LTM) performance. Based on Wischnewski *et al.* (2021) placebo‐controlled effects of theta tACS on LTM were summarized by extracting standardized effect sizes and simulating electric‐field distributions for each study. Specifically, the Hedges' g effect size for tACS (verum–sham) was calculated for LTM outcome measures in each study, based on reported averages in text, tables, or figures. Subsequently, SimNIBS 4.1 was used to simulate electric fields based on the reported tACS montage (Thielscher *et al*. 2015). For modelling, we used a standard head model provided by SimNIBS. Next, all electric field models were loaded into MATLAB 2024a, which provided the electric field strength at each model node. For each node, the vector of electric field values across all studies is correlated with the Hedges' g values, yielding a correlation value per node, known as the performance‐electric field index (PEI). PEI values range between −1 and 1, where positive values suggest that theta tACS improves LTM performance and negative values suggest that theta tACS decreases LTM performance. Note that the results shown here were based on a preliminary analysis of *N* = 17 articles. The completed analysis of *N* = 20 studies is reported in Chitic & Wischnewski (2025), which also contains a more detailed description of inclusion criteria, included studies, and analysis methods. Based on the preliminary data shown here, it was found that the dorsomedial prefrontal cortex (dmPFC) shows the most positive association with improved LTM performance. In contrast, left lateral frontal and temporal regions were negatively associated with LTM performance.
**Figure S2:** Hit rate across conditions. Hit rate scores showed a similar effect compared to the main D‐prime analysis. Significant memorability (F(1,32) = 701,35, *p* < 0.001) and picture category (F(2,64) = 86.46, p < 0.001) effects were found. However, no effect of tACS was observed (F(1, 32) = 0.32, *p* = 0.574). Furthermore, no significant interaction between tACS and memorability (F(1,32) = 0.95, *p* = 0.337), nor picture category (F(2,64) = 2.00, *p* = 0.143), was observed.
**Figure S3:** False alarm rate across conditions. Hit rate scores showed a similar effect compared to the main D‐prime analysis. A significant effect of picture category was found (F(2,64) = 54.63, *p* < 0.001). However, no effect of tACS (F(1, 32) = 0.02, *p* = 0.877), nor of memorability were observed (F(1, 32) = 0.72, *p* = 0.403). The absence of a memorability effect is likely explained by low overall false alarm rates, which are suppressed by a ceiling effect. Furthermore, no significant interaction between tACS and memorability (F(1,32) = 0.70, *p* = 0.502), nor picture category (F(2,64) = 0.62, *p* = 0.438), was observed.

## Data Availability

Data are available on Zenodo (https://doi.org/10.5281/zenodo.17826466).
