# Supplementary material for: Theta Transcranial Alternating Current Stimulation Over the Dorsomedial Prefrontal Cortex Does Not Enhance Long‐Term Memory
Source: Eur J Neurosci. 2026 Feb 10;63(3):e70431. doi: 10.1111/ejn.70431 (PMC12892018; doi:10.1111/ejn.70431)
Supplement: Supplementary file 1 — Figure S1: Meta‐analytic evidence (N = 17) on optimal stimulation location for theta tACS related to long‐term memory (LTM) performance. Based on Wischnewski et al. (2021) placebo‐controlled effects of theta tACS on LTM were summarized by extracting standardized effect sizes and simulating electric‐field distributions for each study. Specifically, the Hedges' g effect size for tACS (verum–sham) was calculated for LTM outcome measures in each study, based on reported averages in text, tables, or figures. Subsequently, SimNIBS 4.1 was used to simulate electric fields based on the reported tACS montage (Thielscher et al. 2015). For modelling, we used a standard head model provided by SimNIBS. Next, all electric field models were loaded into MATLAB 2024a, which provided the electric field strength at each model node. For each node, the vector of electric field values across all studies is correlated with the Hedges' g values, yielding a correlation value per node, known as the performance‐electric field index (PEI). PEI values range between −1 and 1, where positive values suggest that theta tACS improves LTM performance and negative values suggest that theta tACS decreases LTM performance. Note that the results shown here were based on a preliminary analysis of N = 17 articles. The completed analysis of N = 20 studies is reported in Chitic & Wischnewski (2025), which also contains a more detailed description of inclusion criteria, included studies, and analysis methods. Based on the preliminary data shown here, it was found that the dorsomedial prefrontal cortex (dmPFC) shows the most positive association with improved LTM performance. In contrast, left lateral frontal and temporal regions were negatively associated with LTM performance. Figure S2: Hit rate across conditions. Hit rate scores showed a similar effect compared to the main D‐prime analysis. Significant memorability (F(1,32) = 701,35, p < 0.001) and picture category (F(2,64) = 86.46, p < 0.001) effects were [file EJN-63-0-s001.docx]

**Theta transcranial alternating current stimulation (tACS) over DMPFC does not enhance long-term memory**

Dima Chitic^1,2^, Krasimir S. Zdravkov^1^, Vasiliki Dounavi^1^, Felix Grothus^1^, Mark R. Nieuwenstein^1,2^, Miles Wischnewski^1,2^*

**Supplementary Materials**

**
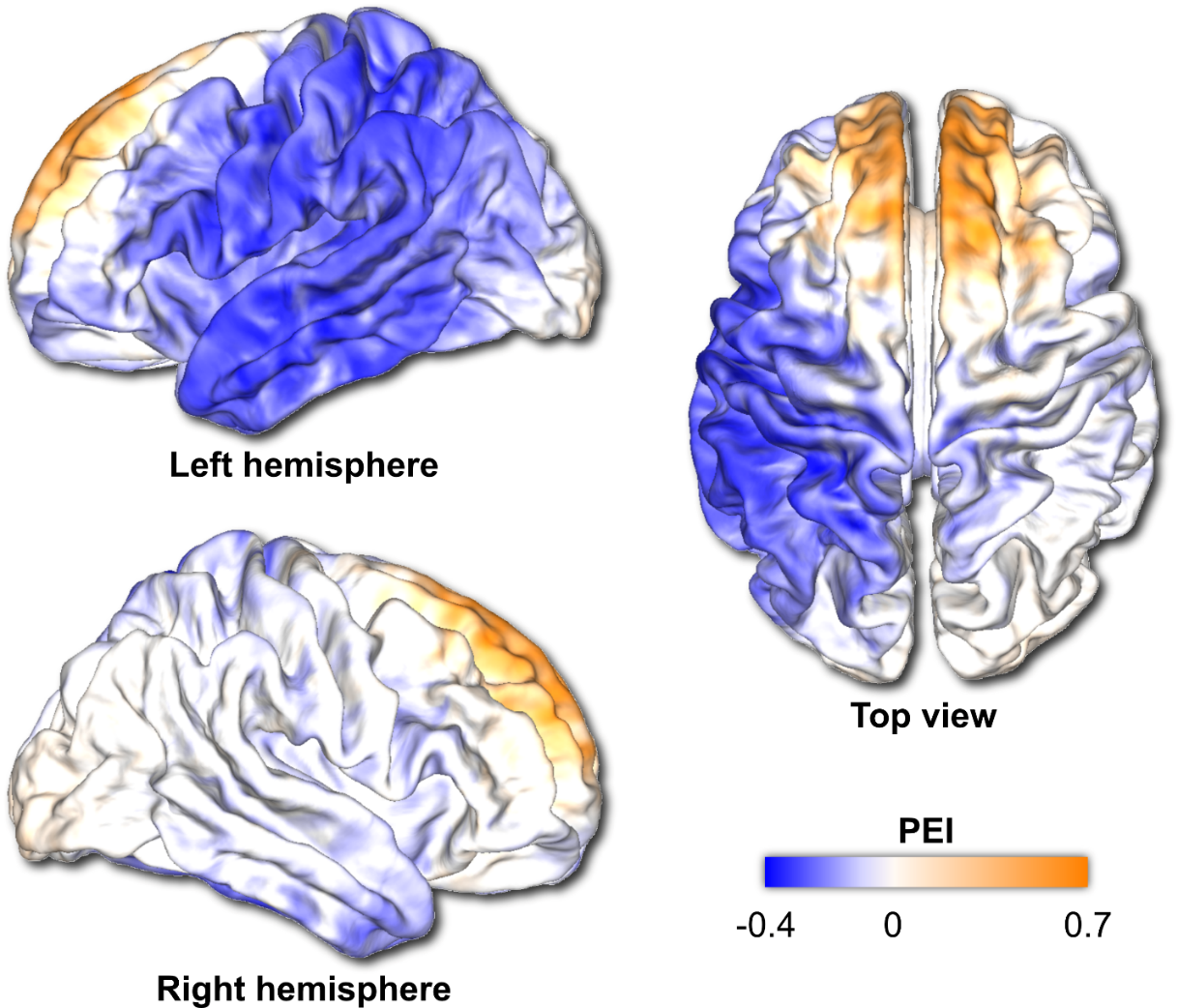
**

**Supplementary Figure 1.** Meta-analytic evidence (N=17) on optimal stimulation location for theta tACS related to long-term memory (LTM) performance. Based on Wischnewski *et al.*, (2021) placebo-controlled effects of theta tACS on LTM were summarized by extracting standardized effect sizes and simulating electric-field distributions for each study. Specifically, the Hedges’ g effect size for tACS (verum–sham) was calculated for LTM outcome measures in each study, based on reported averages in text, tables, or figures. Subsequently, SimNIBS 4.1 was used to simulate electric fields based on the reported tACS montage (Thielscher *et al.*, 2015). For modeling, we used a standard head model provided by SimNIBS. Next, all electric field models were loaded into MATLAB 2024a, which provided the electric field strength at each model node. For each node, the vector of electric field values across all studies is correlated with the Hedges’ g values, yielding a correlation value per node, known as the performance-electric field index (PEI). PEI values range between -1 and 1, where positive values suggest that theta tACS improves LTM performance and negative values suggest that theta tACS decreases LTM performance. Note that the results shown here were based on a preliminary analysis of N=17 articles. The completed analysis of N=20 studies is reported in Chitic & Wischnewski (2025), which also contains a more detailed description of inclusion criteria, included studies, and analysis methods. Based on the preliminary data shown here, it was found that the dorsomedial prefrontal cortex (dmPFC) shows the most positive association with improved LTM performance. In contrast, left lateral frontal and temporal regions were negatively associated with LTM performance.


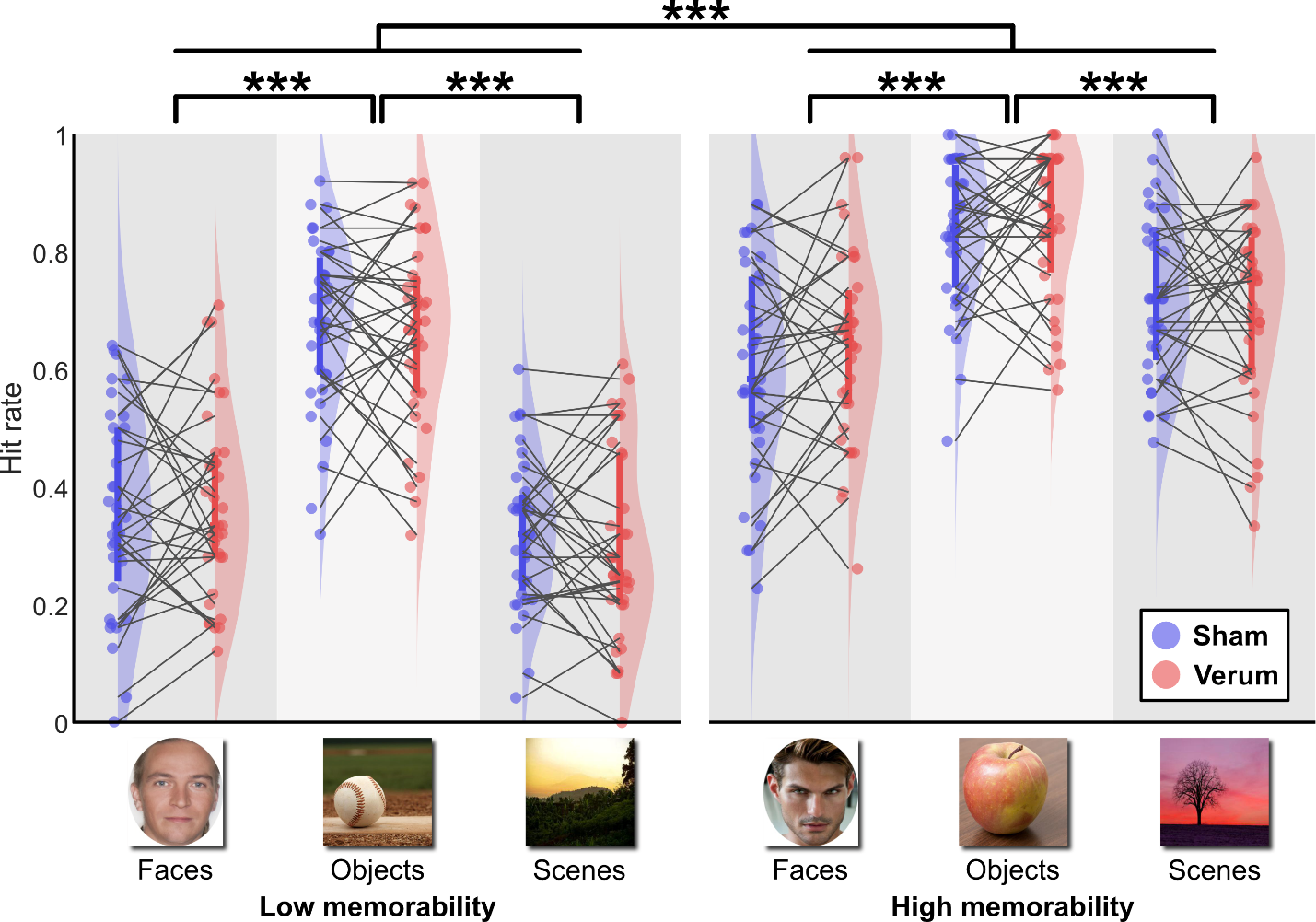


**Supplementary Figure 2.** Hit rate across conditions. Hit rate scores showed a similar effect compared to the main D-prime analysis. Significant memorability (F(1,32) = 701,35, p < 0.001) and picture category (F(2,64) = 86.46, p < 0.001) effects were found. However, no effect of tACS was observed (F(1, 32) = 0.32, p = 0.574). Furthermore, no significant interaction between tACS and memorability (F(1,32) = 0.95, p = 0.337), nor picture category (F(2,64) = 2.00, p = 0.143), was observed.


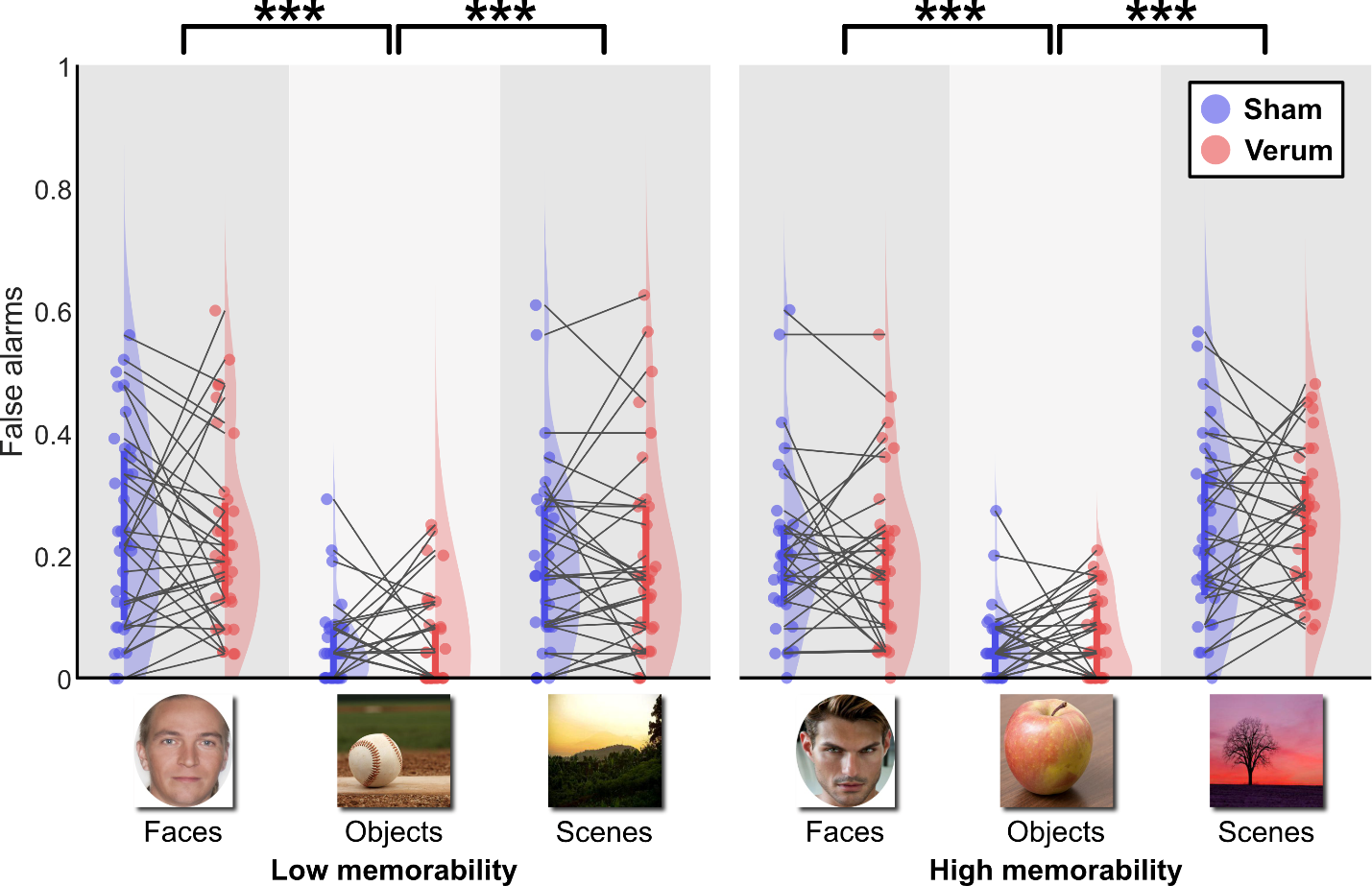


**Supplementary Figure 3.** False alarm rate across conditions. Hit rate scores showed a similar effect compared to the main D-prime analysis. A significant effect of picture category was found (F(2,64) = 54.63, p < 0.001). However, no effect of tACS (F(1, 32) = 0.02, p = 0.877), nor of memorability were observed (F(1, 32) = 0.72, p = 0.403). The absence of a memorability effect is likely explained by low overall false alarm rates, which are suppressed by a ceiling effect. Furthermore, no significant interaction between tACS and memorability (F(1,32) = 0.70, p = 0.502), nor picture category (F(2,64) = 0.62, p = 0.438), was observed.
